# Smart exoskeleton-assisted rehabilitation after fracture surgery: closed-loop control, personalized load management, and integrated telemonitoring

**DOI:** 10.3389/fbioe.2026.1792573

**Published:** 2026-05-26

**Authors:** Tengbo Pei, Zihan Qu, Yutian Lei, Yufang Gao, Tao Xu, Weina Yang, Qifu Wen, Qiang Liu

**Affiliations:** 1 Department of Medical Laboratory, Xianyang Central Hospital, Xianyang, Shaanxi, China; 2 Department of Scientific Research Management, Xianyang Central Hospital, Xianyang, China; 3 Department of Orthopedics I, Xi’an Daxing Hospital, Xi’an, Shaanxi, China; 4 Department of Human Anatomy, Histology and Embryology, School of Basic Medical Sciences, Xi’an Jiaotong University Health Science Center, Xi’an, Shaanxi, China; 5 Department of Orthopedics, Xianyang Central Hospital, Xianyang, Shaanxi, China

**Keywords:** closed-loop control, fracture rehabilitation, personalized load management, smart exoskeleton, telemonitoring

## Abstract

Bone fractures represent a significant global health challenge, particularly with the aging population, where traditional post-operative rehabilitation often falls short of meeting increasingly personalized recovery needs. Smart exoskeleton technology is evolving from passive orthoses toward powered, sensor-rich systems that integrate multimodal sensing—including sEMG, IMUs, and pressure sensors—to enable closed-loop control and dynamic assistance. This review summarizes the system architecture and control strategies of smart exoskeletons in post-fracture rehabilitation, focusing on personalized load management through “assist-as-needed” (AAN) algorithms and progressive loading protocols. Clinical decision-making for exoskeleton use must integrate the patient’s biological condition—such as bone quality, age, and trauma complexity—with surgical fixation stability to aim to indirectly regulate mechanical loading toward the beneficial mechanobiological window of 2%–10% interfragmentary strain. While current evidence suggests these devices are safe and can improve range of motion and hospital stay duration, the clinical base remains heterogeneous, and consistent superiority over conventional therapy for complex fractures is yet to be established. Significant hurdles remain, including high device weight (12–27 kg), cost, anthropometric incompatibility, and the lack of standardized clinical frameworks. Future directions point toward lightweight, adaptive systems enhanced by Digital Twin technology for biomechanical simulation and predictive healing modeling. Furthermore, the integration of telemonitoring and multimodal care pathways will be essential to transition these systems from laboratory settings into routine clinical and home-based care.

## 1 Introduction

### 1.1 Global burden of fractures and unmet needs in post-operative rehabilitation

Bone fractures remain a major global health challenge, with the absolute number of incident and prevalent cases rising due to population aging and increased life expectancy (Wang et al., 2025). In 2019, there were over 178 million new fractures and 455 million people living with fracture-related symptoms worldwide, with the highest rates in older adults and lower limb fractures contributing most to disability (Shen et al., 2022; Tarantino et al., 2022; Khan et al., 2023). Fragility fractures, often linked to osteoporosis, are associated with significant morbidity, mortality, and healthcare costs, particularly in the elderly (Åkesson et al., 2022; Unnanuntana et al., 2023; Silvester et al., 2024). Despite advances in surgical management, post-operative rehabilitation is critical for functional recovery and secondary prevention, yet access and delivery remain inconsistent, especially in low- and middle-income regions (Handoll et al., 2021; Wang and Seibel, 2024; Carter et al., 2025).

Persistent gaps include underdiagnosis and undertreatment of osteoporosis, delayed mobilization, and lack of standardized rehabilitation protocols (Mitchell et al., 2022). Fewer than 20% of patients receive appropriate osteoporosis therapy after an initial fracture, perpetuating the care gap (Åkesson et al., 2022; Tarantino et al., 2022). Rehabilitation interventions are heterogeneous, with limited reporting on adherence and poor integration of technology (Richardson et al., 2022; Bretherton et al., 2024). The increasing complexity of fracture patterns and comorbidities in aging populations underscores the need for innovative, scalable, and personalized rehabilitation strategies, such as exoskeleton-assisted therapy and telemonitoring, to improve outcomes and address these unmet needs (Handoll et al., 2021; Sarkies et al., 2023; Carter et al., 2025).

### 1.2 Evolution from passive orthoses to powered, sensor-rich exoskeletons

The development of exoskeleton technology for rehabilitation has progressed from passive orthoses, which provide static mechanical support, to powered, sensor-rich systems capable of dynamic, adaptive assistance. Early orthoses, such as knee-ankle-foot orthoses (KAFOs), were primarily passive devices designed to stabilize joints and enable basic ambulation, but they often resulted in compensatory gait patterns and limited functional gains (Rodríguez-Fernández et al., 2022). The limitations of passive devices, including lack of adaptability and user-specific customization, prompted the integration of actuators and control systems, giving rise to active exoskeletons that can deliver joint-specific torque and facilitate more physiological movement patterns (Chen et al., 2023a; Siviy et al., 2023; Daliri et al., 2024).

Modern powered exoskeletons incorporate a range of sensors—such as force, displacement, inertial measurement units, and electromyography (EMG)—to monitor user intent and biomechanical state in real time, enabling closed-loop control and personalized assistance (Rosales-Luengas et al., 2023; Yao et al., 2024). These sensor-rich systems can detect subtle changes in muscle activation or load distribution, allowing for adaptive modulation of support during complex tasks like sit-to-stand transitions or variable-speed walking (Yao et al., 2024). Advances in control algorithms, including those leveraging artificial intelligence, have further enhanced the responsiveness and safety of exoskeletons, supporting individualized rehabilitation protocols and optimizing outcomes for diverse patient populations (Siviy et al., 2023; Boyapati et al., 2025).

The transition from rigid, one-size-fits-all devices to soft, compliant, and modular exoskeletons has addressed many user-identified barriers, such as comfort, weight, and ease of donning, while also expanding the range of clinical applications (Morris et al., 2023; Gavrila Laic et al., 2024). Despite these advances, challenges remain in achieving seamless human-exoskeleton interaction, enhancing device reliability, and translating laboratory innovations into routine clinical practice (Chen et al., 2023a; Siviy et al., 2023). Ongoing research continues to refine sensor integration, actuation strategies, and user-centered design to maximize the therapeutic potential of exoskeleton-assisted rehabilitation.

### 1.3 Aim and scope of this review

**FIGURE 1:**
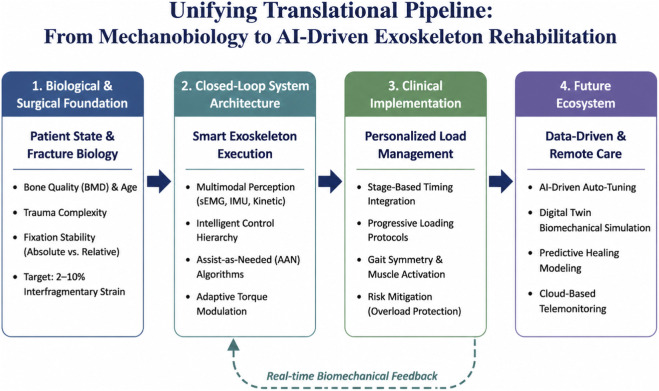
Unifying conceptual framework and translational pipeline for smart exoskeleton-assisted fracture rehabilitation. This high-level overview illustrates the progression from basic mechanobiological principles to advanced clinical ecosystems. The pipeline begins with the biological and surgical foundation (Pillar 1), which directly informs the closed-loop system architecture and assist-as-needed algorithms (Pillar 2). These engineering tools are subsequently translated into personalized clinical implementation strategies (Pillar 3), guided by real-time biomechanical feedback. Finally, the framework points toward a future data-driven ecosystem (Pillar 4) enhanced by AI auto-tuning, digital twin simulations, and telemonitoring.

This article aims to establish a unifying conceptual framework for smart exoskeleton-assisted fracture rehabilitation, bridging the gap between biological constraints and engineering execution. Instead of presenting hardware and clinical aspects in isolation, our narrative thread follows a coherent translational pipeline: we begin with the biological foundation of fracture healing (mechanobiological loading windows), transition into the engineering architecture of closed-loop exoskeletons (multimodal sensing and adaptive control), and culminate in the clinical decision-making logic required for personalized load management. Furthermore, we synthesize current clinical evidence, systematically outline key translational barriers (e.g., system constraints, human-robot interaction, and cost), and propose a future roadmap leveraging Digital Twin technology and telemonitoring. Ultimately, this review seeks to provide a comprehensive framework for transitioning these advanced systems from laboratory concepts to routine, data-driven clinical care (Figure 1).

This article is structured as a narrative review. To ensure methodological transparency, literature for this review was primarily retrieved from PubMed, Web of Science, and Scopus databases, covering the period from January 2013 to May 2026. The search strategy utilized combinations of keywords including ‘smart exoskeleton’, ‘fracture rehabilitation’, ‘closed-loop control’, ‘mechanobiology’, and ‘orthopedics’. Initial screening prioritized studies reporting system architectures, clinical trial outcomes, and biomechanical models relevant to post-fracture recovery.

Given the interdisciplinary nature of this review, key terminologies are strictly standardized to maintain conceptual consistency across both engineering and clinical domains. Closed-loop control refers specifically to the real-time adjustment of exoskeleton actuator output based on continuous feedback from biomechanical or physiological sensors. Assist-as-needed (AAN) is operationally defined as the real-time inverse modulation of robotic torque relative to the user’s voluntary active effort. Personalized load management denotes the strategic regulation of mechanical stress—specifically, aiming to indirectly regulate loading toward the optimal interfragmentary strain window—tailored to the patient’s evolving fracture stability and physiological healing stage.

## 2 System architecture for closed-loop fracture rehabilitation

Closed-loop exoskeletons enable patient-specific fracture rehabilitation through real-time sensing, adaptive control, and dynamic actuation. A unified architecture—comprising biomechanical interfaces, multimodal sensors, actuators, and control algorithms—supports safe and precise postoperative assistance (Yao et al., 2024).

### 2.1 Biomechanical interface and actuation

The biomechanical interface constitutes the critical physical link between the exoskeleton and the patient, requiring anatomical conformity, adjustable fixation, and reduced shear forces to promote safety and comfort (Siviy et al., 2023). Contemporary designs favor modular, lightweight materials to balance rigidity, wearability, and portability (Hussain et al., 2021). Beyond materials, structural paradigms range from rigid linkage-based frames for stable load transfer to soft, cable-driven exosuits that minimize inertia (Rodríguez-Fernández et al., 2021; Siviy et al., 2023). A core mechanical challenge in orthopedics is achieving precise kinematic alignment between the device and biological joints to avoid pathological shear forces (Bessler-Etten et al., 2022). To address this, novel mechanisms such as the Coupled Movable Pulley Mechanism (CMPM) have been proposed to optimize torque transmission and enhance alignment compatibility (Ji et al., 2024).

Actuation technologies now extend from conventional motors to compliant systems such as series elastic, pneumatic, and flexible actuators, improving safety and accommodation of nonlinear joint mechanics (Helian et al., 2025). Actuator choice is closely tied to rehabilitation stage, ranging from high-torque passive mobilization to compliant, patient-driven training (Hussain et al., 2021).

Sensor-driven adaptive control algorithms dynamically regulate actuator output to enforce safety limits and enable assist-as-needed and progressive loading strategies throughout bone healing (Siviy et al., 2023; Yao et al., 2024).

Beyond conventional full-limb exoskeletons, a comprehensive orthopedic rehabilitation paradigm must incorporate a broader spectrum of specialized systems tailored to specific fracture types and healing stages. For instance, body-weight-support (BWS) systems and mobile robotic platforms (e.g., SWalker) are critical in early-stage rehabilitation, particularly for frail patients with hip fractures, facilitating safe overground ambulation while effectively offloading vulnerable joints (Costa et al., 2022). As patients progress, smart orthoses bridge the gap between passive stabilization and active assistance. These devices integrate flexible or electrospinning sensors to continuously monitor joint kinematics and provide variable resistance without the bulk of fully powered systems (Lee et al., 2025). Furthermore, for periarticular injuries where early mobilization is vital to prevent stiffness, joint-specific postoperative rehabilitation robots (such as specialized robotic devices for elbow or knee recovery) provide localized, high-precision trajectory control to safely restore range of motion (Helian et al., 2025). Collectively, incorporating these diverse fracture-related exoskeletons and orthopedic rehabilitation robots expands the clinical toolkit, enabling more granular, patient-specific load management.

**FIGURE 2:**
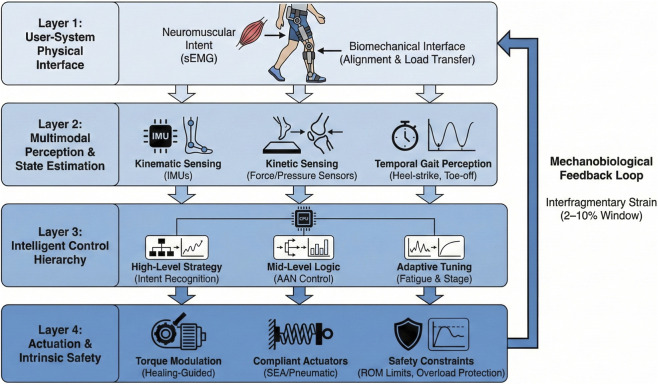
Hierarchical Architecture for Closed-Loop Smart Exoskeleton-Assisted Fracture Rehabilitation. The system is organized into four functional layers to enable personalized recovery. Layer 1 (Human-Machine Physical Interface) establishes the physical and biological connection, capturing neuromuscular intent via sEMG and facilitating anatomical alignment. Layer 2 (Multimodal Perception & Sensor Fusion) integrates kinematic (IMUs) and kinetic (pressure/force) data to recognize gait phases and estimate biomechanical states in real time. Layer 3 (Intelligent Control Hierarchy) serves as the decision-making core, utilizing “Assist-as-Needed” (AAN) logic and adaptive tuning to modulate assistance based on the patient’s residual function and healing progress. Layer 4 (Actuation & Execution) delivers regulated torque through compliant actuators while enforcing intrinsic safety constraints to prevent joint overload. The global feedback loop aims to indirectly regulate mechanical loading to approximate the therapeutic window (2%–10%) to optimize bone regeneration.

Together, these elements define a unified closed-loop architecture for safe and personalized fracture rehabilitation (Figure 2). To provide a structured summary of available quantitative and qualitative information, Table 1 outlines the core mechanical and structural design parameters for representative systems.

**TABLE 1:** Structured summary of available design parameters for representative rehabilitation exoskeletons.

Representative system	Structural Configuration	Actuation type	Mass (kg)	Active DoFs (per leg)	Nominal output capability	Reference
CMPM Prototype	Rigid linkage with specialized pulleys	Cable-driven (coupled movable pulley)	Not specified (lightweight design)	2 (Hip, Knee)	Optimized non-linear torque transmission	Ji et al. (2024)
SWalker	Mobile robotic platform/Hybrid	Overground body-weight support motors	N/A (Wheeled platform)	Pelvic/Trunk stabilization	Dynamic weight unloading, fall prevention	Costa et al. (2022)
Ekso Bionics (EksoGT)	Rigid frame, anthropomorphic	Brushless DC electric motors	23	2 (Hip, Knee)	Variable assistance, up to 100% gait power	Bach Baunsgaard et al. (2018)
ABLE Exoskeleton	Modular, lightweight rigid frame	Frameless BLDC electric motors	10	1 or 2 (Knee/Hip)	Active knee/hip extension and flexion assist	Wright et al. (2023)
Soft Exosuits (General Paradigm)	Soft textiles, cable-driven	Bowden cables with remote actuators	<5.0 (worn mass)	1 to 2 (Ankle/Hip)	Low-inertia joint torque assistance	Siviy et al. (2023)
Smart Orthosis	Lightweight functional brace with embedded flexible sensors	Passive/Semi-active	<2.0	1 (e.g., Knee/Ankle)	Kinematic monitoring, variable damping	Lee et al. (2025)
Joint-Specific Postoperative Robot	Single-joint specialized modular frame	Direct drive/Cable-driven	Varies	1 (e.g., Elbow/Knee specific)	High-precision continuous passive motion and active-assist	Helian et al. (2025)

This is a structured summary of available literature. Qualitative descriptions are used where exact quantitative metrics were not reported.

### 2.2 Sensing and state estimation

Accurate sensing and state estimation underpin closed-loop exoskeleton-assisted fracture rehabilitation. Closed-loop feedback is enabled by multimodal sensing at the interface, including sEMG, force/torque, and plantar pressure sensors, which capture muscle activation, joint loading, and weight-bearing patterns in real time (Yao et al., 2024). These signals support intent recognition and adaptive assistance during rehabilitation (Mosconi et al., 2024).

Specifically, IMUs capture limb kinematics for activity recognition, gait analysis, and force estimation, with machine learning further enhancing intent detection and individualized control (Matijevich et al., 2020). Force sensors at joints and contact interfaces monitor loading conditions, enabling safe, adaptive assistance and progressive rehabilitation (Morris et al., 2023; Yao et al., 2024).

Surface electromyography (EMG) provides muscle-level information for intent-driven and assist-as-needed control, with advanced signal processing improving motion classification and fatigue detection (Rosales-Luengas et al., 2023; Sul et al., 2025). Additional physiological sensors, such as ECG and skin temperature, may supplement safety monitoring in selected populations (Szabo et al., 2023).

Multimodal fusion of kinematic, kinetic, and EMG data enables comprehensive state estimation and effective closed-loop control for personalized rehabilitation (Rosales-Luengas et al., 2023; Yao et al., 2024).

### 2.3 Intrinsic safety by design

Intrinsic safety in rehabilitation exoskeletons requires multilayer risk control across design, control, materials, and human–machine interaction (Mosconi et al., 2024). Assistance and resistance should match patient dynamics to avoid over- or under-support, while real-time sensing (EMG, force, angle) enables rapid detection of abnormal motion and improves control precision (Tiboni et al., 2022).

Ergonomic joint alignment and optimized load-transfer pathways are essential to minimize discomfort and prevent secondary injury from misalignment (Boyapati et al., 2025). Lightweight yet durable materials, such as polymer composites or medical-grade metals, improve comfort and reliability while reducing mechanical failure risk (Vélez-Guerrero et al., 2021b; Massardi et al., 2023).

Safety-oriented control should integrate protection modes, motion-threshold limits, and anomaly detection, enabling automatic shutdown or adaptive adjustment during unexpected events (Zhang et al., 2025). Modeling and simulation further support safe torque and motion-range settings to avoid joint overload or compression (Rangan et al., 2022; Rodríguez-Fernández et al., 2025).

Standardized risk-assessment systems are required to identify hazards such as unintended motion, malalignment, or skin injury, together with preventive measures (Massardi et al., 2023). Clinical evidence suggests modern exoskeletons may offer improved safety and usability than conventional orthoses, with fewer adverse events (Bao et al., 2019; Rodríguez-Fernández et al., 2025).

With AI and remote monitoring, personalized load management and closed-loop safety control are expected to further enhance outcomes. Overall, intrinsic safety should span the full lifecycle from engineering design to clinical application.

## 3 Closed-loop control strategies

Closed-loop control is a core component of smart exoskeleton systems, enabling real-time adjustment of assistance based on user-specific biomechanics and sensor feedback (de Miguel-Fernández et al., 2023). This section outlines key algorithmic strategies used to detect gait phases and deliver personalized, phase-specific support during rehabilitation (Lee et al., 2024).

### 3.1 State-aware control strategies

Reliable state perception relies on multisensor fusion—such as combining IMUs, pressure sensors, and EMG—to optimize gait-phase detection and intention prediction, with a structured summary of available functional and performance indicators detailed in Table 2 (Guerra et al., 2024; D’Arco et al., 2025; Lee et al., 2025).

**TABLE 2:** Structured summary of representative closed-loop control strategies and reported performance indicators.

Control strategy	Key performance indicator/Tracking error	Response latency/Timing	Interaction performance & stability	Primary application & phase	Reference
Multisensor State-Machine (IMU + Pressure)	<7% gait-phase detection error	Real-time estimation	Highly stable across various walking speeds and loads	Gait-phase recognition	Guerra et al. (2024), D’Arco et al. (2025)
EMG-based Intention Prediction	Highly dependent on signal clarity	∼120 m in advance	Enhances human-machine synchronization	Early motor intent detection	Guerra et al. (2024)
Impedance/Compliance Control	Real-time tracking of dynamic setpoints	Continuous adaptation	Promotes active participation, reduces interaction forces	Assist-as-needed (AAN) training	Chen et al. (2023b), Zhao et al. (2025)
Disturbance Observer-based Control	Significantly improves tracking accuracy	Rapid disturbance rejection	Compensates for dynamic modeling errors, highly stable	Complex/Uncertain biomechanical states	Brahmi et al. (2021)
Interaction-Torque/AAN Control	Optimized to user’s voluntary effort	Real-time torque adjustment	Automatically tunes support to protect repair tissue	Progressive loading across healing stages	Wang et al. (2022), Li et al. (2026a)

This is a structured summary of available literature. Qualitative descriptions are used where exact quantitative metrics were not reported.

Impedance/compliance control provides “assistance-as-needed” by adjusting stiffness and damping in real time, promoting active participation and reducing interaction forces (Chen et al., 2023b; Zhao et al., 2025). Adaptive tuning based on gait uncertainty, muscle strength, or biomechanical feedback enhances safety (Srisuchinnawong et al., 2024). Extended-state or disturbance observers further compensate modeling errors to improve control accuracy (Brahmi et al., 2021).

Clinical studies indicate that state-aware gait detection combined with impedance/compliance control improves synchronization and reduces tracking error, supporting individualized rehabilitation (de Miguel-Fernández et al., 2023). However, large-scale standardized trials are still needed to advance clinical translation (Srisuchinnawong et al., 2024).

### 3.2 Stage-dependent adaptive assistance and progressive loading

In postoperative fracture rehabilitation, closed-loop control in exoskeletons centers on adaptive assistance, where support intensity is dynamically adjusted according to healing stage and functional recovery (de Miguel-Fernández et al., 2023; Delgado and Yihun, 2023). This is enabled by real-time sensing (e.g., EMG, force, IMU) and multimodal data fusion to estimate user participation and physiological status (Baud et al., 2021; Vélez-Guerrero et al., 2021a). Machine-learning and neuromusculoskeletal models further improve intent recognition, torque prediction, and uncertainty handling (Yao et al., 2024).

The progression of assistance must closely align with the biological healing timeline. Early after surgery, higher assistive torque (or low-intensity user modes) is applied to reduce stress at the fracture site and protect the repair tissue (de Miguel-Fernández et al., 2023; Delgado and Yihun, 2023). As healing progresses, feedback control gradually reduces assistance, thereby safely increasing voluntary loading to promote muscle activation and functional recovery (Siviy et al., 2023; Boyapati et al., 2025). This progressive loading relies on continuous assessment of strength, range of motion, and pain (Salgado-Gomes-Sagaz et al., 2024).

Algorithmic execution of this progression is achieved through on-demand assistance and interaction-torque control (Wang et al., 2022; Delgado and Yihun, 2023). Force- and EMG-based approaches quantify residual function and adapt support levels, automatically tuning the robotic output based on real-time muscle activity and joint dynamics (Vélez-Guerrero et al., 2021a; Li et al., 2026a). Combining this progressive loading with on-demand assistance has been shown to enhance rehabilitation outcomes in both acute and chronic phases (de Miguel-Fernández et al., 2023; Yao et al., 2024). However, challenges remain in standardization and individualized parameter tuning, requiring continued algorithm refinement and multicenter validation (Baud et al., 2021).

To bridge the gap between engineering control and clinical orthopedics, these key mechanisms are operationally defined as follows. AAN intensity scaling involves real-time inverse torque modulation relative to voluntary muscle output; the exoskeleton reduces assistance when sensors detect sufficient effort and supplies torque only when output drops (Wang et al., 2022; Delgado and Yihun, 2023). Safety-related loading thresholds act as programmable constraints capping peak compressive forces to indirectly regulate mechanical loading toward the 2%–10% interfragmentary strain window (Augat et al., 2021; Zhang et al., 2025). Finally, the mapping of healing stages to control is a time-dependent progression from early-stage high-torque protection to late-stage low-impedance stimulation, aiming to keep assistance subordinate to the structural integrity of the healing bone (de Miguel-Fernández et al., 2023; Boyapati et al., 2025).

Overall, closed-loop adaptive assistance—supported by multimodal sensing and intelligent control—enables progressively loaded and individually tailored support after fracture surgery, improving mobility, safety, and patient engagement.

### 3.3 Synthesis of design and control trade-offs

Exoskeleton selection in post-fracture care involves inherent trade-offs between mechanical stability and functional mobility, representing a critical decision point in clinical implementation. Rigid systems equipped with predefined trajectory control provide absolute kinematic stability. This facilitates reliable joint protection and indirect regulation of interfragmentary strain, which is paramount for early-stage complex fractures. However, their structural bulk, fixed movement patterns, and added inertia can restrict natural gait variability and induce compensatory biomechanics (Bessler-Etten et al., 2022).

Conversely, lightweight compliant exosuits utilizing assist-as-needed (AAN) control paradigms prioritize human-machine transparency. By delivering targeted torque without rigid constraints, they promote active neuromuscular engagement, preserve natural muscle synergies, and prevent disuse atrophy during later healing stages. Yet, these flexible architectures lack the structural rigidity required to provide sufficient mechanical offloading or resist pathological shear forces in patients with unstable fixations (Wang et al., 2022; Siviy et al., 2023). Therefore, optimizing rehabilitation outcomes requires a dynamic approach. Clinicians must continuously match these engineering trade-offs to the patient’s evolving mechanobiological needs across the recovery timeline—transitioning from maximum hardware protection to functional independence (Li et al., 2026a).

## 4 Clinical use cases and decision logic

### 4.1 Mechanical context (load-bearing vs. support)

Load-bearing and assistive support represent two distinct biomechanical strategies in exoskeleton-assisted fracture rehabilitation, selected according to fracture stability and healing stage (Hohl et al., 2022). Load-bearing exoskeletons redistribute ground reaction forces through the device framework, thereby reducing compressive stress on healing bone while maintaining upright gait (De Carvalho et al., 2025). Computational analyses show peak compressive forces of ∼2.98–4.66 BW at the hip, 2.82–5.83 BW at the knee, and 3.39–3.79 BW at the ankle during exoskeleton-assisted walking, providing quantitative guidance for risk assessment in compromised bone (De Carvalho et al., 2025).

Assistive-mode devices instead apply variable torque to support movement while preserving physiological loading patterns (de Miguel-Fernández et al., 2023). Most lower-limb systems use rule-based, ground-reaction-force-triggered assistive control to modulate torque across gait phases (de Miguel-Fernández et al., 2023). This maintains proprioception and neuromuscular engagement, with EMG data indicating that fully passive support may paradoxically increase muscle activation compared with unassisted walking (Mosconi et al., 2024).

Mode selection is driven by fracture characteristics, fixation stability, and time-course of healing (Dehghan et al., 2018). After ankle fracture surgery, immediate weight-bearing in a walking boot yields functional outcomes comparable—or superior—to delayed protocols, with similar complication rates (Khojaly et al., 2025). The WAX trial confirmed that weight-bearing from 2 weeks postoperatively improves early function and lowers healthcare costs compared with non-weight-bearing strategies (Bretherton et al., 2024). In contrast, peri-articular fractures such as tibial plateau, plafond, calcaneal, and acetabular injuries typically require protected loading for 6–8 weeks to prevent joint collapse and implant failure (Kubiak et al., 2013), whereas most hip fracture fixations permit immediate weight-bearing in the elderly (Richardson et al., 2022).

**FIGURE 3:**
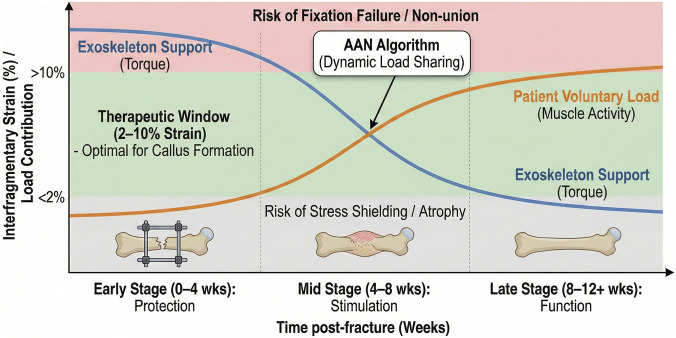
Mechanobiological Window-Driven Progressive Loading and Exoskeleton Assistance Protocol. This figure illustrates the synchronization of exoskeleton assistance with fracture healing phases. Initially, high assistive torque protects the fracture site during the inflammatory stage. During callus formation, the system aims to indirectly regulate interfragmentary strain toward the 2%–10% therapeutic window using “assist-as-needed” (AAN) logic to stimulate bone regeneration. Finally, assistance is tapered to focus on functional recovery and gait symmetry, supporting stage-specific mechanical loading for optimal healing.

Timing and magnitude of loading remain critical mechanobiological determinants of healing (Figure 3). Early loading during days 0–7 may disrupt hematoma organization (Augat et al., 2021), whereas controlled loading during weeks 2–4 enhances callus formation and promotes osteogenesis (Liu et al., 2018). Beneficial interfragmentary strain generally lies within 2%–10% (Augat et al., 2021), with spatial strain gradients directing tissue differentiation (Liu et al., 2018). Progressive loading protocols exploit these responses to stimulate bone regeneration while maintaining safety thresholds (Anani and Castillo, 2022). Crucially, this differentiates orthopedic applications from neurorehabilitation (e.g., stroke or spinal cord injury). While neurorehabilitation prioritizes restoring motor control and muscle activation (de Miguel-Fernández et al., 2023), exoskeleton assistance in fracture care must remain subordinate to the structural integrity of the healing bone and fixation hardware (Kubiak et al., 2013). Consequently, any applied torque must not exceed hardware thresholds, aiming to indirectly regulate mechanical loading to support this therapeutic window to prevent implant failure or delayed union (Augat et al., 2021; Anani and Castillo, 2022). Clinically, patient-specific frameworks integrating fracture biology, device capability, and supervised training are essential, as exoskeletons can shorten rehabilitation duration—as shown with the SWalker platform in elderly hip-fracture patients—yet remain limited by setup complexity and device burden (Ehrlich-Jones et al., 2021).

### 4.2 Biological context (bone quality, age, trauma complexity)

Smart exoskeleton rehabilitation after fracture surgery should be aligned with the patient’s biological condition, with bone quality, age, and trauma complexity guiding device selection, control strategy, and safety thresholds (Li et al., 2026a). Bone mineral density (BMD) is critical: patients with osteoporosis or low BMD face higher risk of fragility fractures during weight-bearing exoskeletal training, requiring rigorous screening to prevent device-related injury (Boyapati et al., 2025). Age-related changes, including sarcopenia and reduced intrinsic capacity, influence both safety and efficacy, with older adults benefiting from lower-intensity, support-focused programs and telemonitoring to reduce sedentary behavior and optimize recovery (Gavrila Laic et al., 2024; Spungen et al., 2024).

Trauma complexity, such as comminuted or periarticular fractures, necessitates individualized load management, since excessive mechanical strain may disrupt healing or compromise fixation stability (Siviy et al., 2023). Closed-loop control systems using real-time multimodal feedback (e.g., EMG, force, displacement sensors) enable dynamic adjustment of assistance based on patient-specific biomechanical and biological signals, supporting safe rehabilitation progression (Yao et al., 2024). In patients with severe neuromotor deficits or complex injuries, exoskeletons can augment lower-limb strength and balance, though gait outcomes do not consistently surpass conventional therapy, highlighting the value of combined or hybrid approaches (Chang et al., 2025; Liu et al., 2025).

Telemonitoring platforms allow remote assessment of bone health, adherence, and adverse events, particularly in elderly or high-risk patients, supporting early intervention and reducing hospitalization (Vita et al., 2025). Integrating personalized load management, closed-loop control, and telemonitoring enables biologically informed exoskeleton rehabilitation that accommodates variability in bone quality, age, and trauma complexity after fracture surgery.

### 4.3 Surgical context (fixation stability)

**FIGURE 4:**
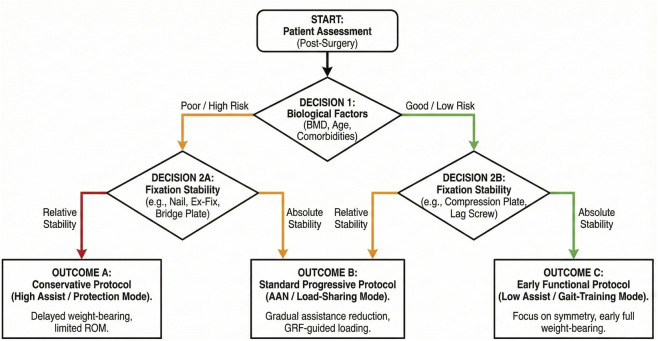
Multidimensional Clinical Decision Algorithm for Exoskeleton-Assisted Fracture Rehabilitation. This algorithm integrates surgical, biological, and mechanical variables to guide device selection and loading protocols. The decision logic first assesses fixation stability—distinguishing between absolute stability for early mobilization and relative stability for protected loading—while accounting for trauma complexity. It further incorporates biological factors such as age and bone mineral density (BMD) to mitigate risks in osteoporotic or geriatric populations. The final output determines the optimal exoskeleton mode (load-bearing vs. assistive support) and the timing of progressive weight-bearing, designed to keep the loading remains within safe biomechanical thresholds throughout the healing process.

Fixation stability is the key determinant of postoperative weight-bearing and rehabilitation timing, as construct integrity sets the safe loading threshold during healing (Figure 4). Stable fixation permits earlier mobilization, while instability risks reduction loss or implant failure; therefore, loading tolerance must consider fracture pattern, implant design, reduction quality, and bone stock (Kubiak et al., 2013). These concepts define absolute versus relative stability, with compression plating enabling primary healing and bridge plating or intramedullary nailing supporting callus formation (Norris et al., 2018). Although their mechanics differ, both nails and locking plates achieve reliable union when appropriately selected (Brodke et al., 2025), while locking technology acts as an internal fixator and provides enhanced stability in osteoporotic or comminuted bone (Le Baron et al., 2024).

Construct biomechanics and fracture complexity govern loading capacity. Locked plates show greater stiffness in metaphyseal and periarticular fractures and outperform nails when lateral support is absent (Norris et al., 2018), whereas intramedullary devices offer load-sharing advantages in diaphyseal fractures (Bretherton et al., 2024). Stable intertrochanteric fractures generate lower implant and cancellous bone stress than unstable types, and cephalomedullary nails reduce stress compared with sliding hip screws in unstable patterns (Kyriakopoulos et al., 2022). Yet surgeon judgment remains variable and subjective, with most weight-bearing recommendations based on perceived stability rather than standardized criteria (Granlund and von Keudell, 2025).

Growing evidence supports earlier weight-bearing following stable fixation. Trials in ankle fractures show early or immediate loading yields equivalent or superior outcomes without increased complications (Bretherton et al., 2024). Similar results are reported for selected tibial plateau fractures treated with locked plating, with no fixation failures despite full loading (Williamson et al., 2018). Elderly fragility fracture patients frequently mobilize immediately, particularly after hip fixation (Richardson et al., 2022). Overall, when fixation stability is achieved, early mobilization shortens hospital stay without added risk, while high-risk articular injuries still warrant caution.

Building upon these biological and surgical considerations, Table 3 provides a structured framework for operational patient stratification. By categorizing rehabilitation risk based on fixation stability, bone quality, fracture complexity, and healing stage, this matrix allows clinicians to map specific patient profiles to appropriate exoskeleton operational strategies.

**TABLE 3:** Structured framework for operational patient stratification in exoskeleton-assisted rehabilitation.

Rehabilitation risk	Clinical & surgical profile	Healing stage	Recommended operational strategy	Supporting references
Low Risk	Fixation: Absolute stability (e.g., plate) Bone Quality: Normal BMD, younger Complexity: Simple/stable fractures	Mid-stage (callus formation, weeks 2–6)	Assistive/AAN Mode: Focus on gait symmetry and early progressive loading	Bretherton et al. (2024), Khojaly et al. (2025), Xu et al. (2026)
Moderate Risk	Fixation: Relative stability (e.g., nail) Bone Quality: Osteopenia, older age Complexity: Comminuted but aligned	Mid to late-stage (soft to hard callus, weeks 4–12)	Hybrid Mode: Adaptive torque to indirectly regulate therapeutic strain	Fu et al. (2023), Li et al. (2026b)
High Risk	Fixation: Poor/Unstable fixator Bone Quality: Severe osteoporosis, frail Complexity: Periarticular or bone loss	Early to mid-stage (inflammatory to early callus, weeks 0–6)	Protection Mode: Rigid offloading and restricted range of motion (ROM)	Hohl et al. (2022), Liang et al. (2024)

This framework represents a structured synthesis of available clinical guidelines and biomechanical evidence. Operational strategies should be adapted based on individual patient progress and specific surgical constraints.

## 5 Evidence base and outcome domains

### 5.1 Clinical evidence overview

The clinical evidence for exoskeleton-assisted rehabilitation after fracture surgery remains limited, with most research focusing on neurological rather than orthopedic conditions (Hohl et al., 2022). Given this scarcity of orthopedic-specific trials, a substantial portion of the technical and safety evidence cited in this review is derived from neurorehabilitation studies (Hohl et al., 2022). We retain this evidence because fundamental human-machine interaction principles—such as baseline device safety, prevention of skin contact lesions, ergonomic joint alignment, and standard gait-synchronization control algorithms—are highly transferable to fracture patients (Bach Baunsgaard et al., 2018; Wright et al., 2023). However, we explicitly emphasize that applying these technologies to orthopedic populations requires superimposing the aforementioned stage-dependent healing and fixation stability constraints onto these baseline neurological algorithms (Siviy et al., 2023). Although several FDA-approved exoskeleton systems are available, there is little guidance regarding optimal device selection, initiation timing, or implementation strategies in routine practice (Hohl et al., 2022). Systematic reviews of wearable lower-limb exoskeletons show that current evidence is largely derived from small, short-term trials with heterogeneous protocols, indicating that these technologies remain in an early developmental phase (Rodríguez-Fernández et al., 2021).

Orthopedic-specific applications demonstrate emerging but inconsistent benefits (Quacinella et al., 2019). In patients with pilon fractures, the Intrepid Dynamic Exoskeletal Orthosis (IDEO) produced only modest gains in gait velocity (1.1–1.3 m/s, p = 0.01) without meaningful improvements in cadence, stride length, stance time, or pain (Quacinella et al., 2019). As direct orthopedic evidence, following total knee arthroplasty, a meta-analysis of eight studies (302 patients) reported that exoskeletons significantly improved active and passive range of motion (SMD 10.98 and 4.11, respectively), Hospital for Special Surgery scores (SMD 7.78), and reduced hospital stay length (SMD −3.19) compared with conventional rehabilitation (Wu et al., 2024).

Overall safety profiles suggest exoskeleton training is generally well tolerated (Bach Baunsgaard et al., 2018; Louie et al., 2021). Conversely, relying on indirect evidence from neurorehabilitation, one study of 52 spinal cord injury patients undergoing 8 weeks of training, no serious adverse events occurred, although several participants developed transient ankle swelling or Category II pressure ulcers at device contact points (Bach Baunsgaard et al., 2018). An umbrella review of 62 systematic reviews including 341 randomized trials and 14,522 participants concluded that robot-assisted training improves multiple outcomes across diseases without major safety concerns (Kiyono et al., 2024). Despite these favorable general safety profiles, a critical evidence gap specific to the post-fracture population remains: the absence of head-to-head randomized controlled trials comparing the clinical efficacy of different exoskeleton designs (e.g., rigid vs. soft architectures) across distinct fracture patterns.

### 5.2 Outcome domains used to evaluate efficacy

Outcome domains evaluating the efficacy of smart exoskeleton-assisted rehabilitation after fracture surgery include functional performance, patient-reported outcomes, neuromuscular activation, gait quality, pain, and safety (Gavrila Laic et al., 2024). Functional performance is typically measured using standardized mobility assessments such as the Timed Up and Go, Five Times Sit-to-Stand, and 10-m Walk Test, which sensitively capture improvements in balance and ambulation following exoskeleton use (Vita et al., 2025). Patient-reported outcome measures (PROMs), including PROMIS Physical Function and Pain Interference, the Disabilities of the Arm, Shoulder and Hand (DASH), and the Lower Extremity Functional Scale (LEFS), provide validated indicators of physical function, pain, and participation after fracture surgery (Havermans et al., 2023).

Neuromuscular activation is quantified through electromyography and torque assessment to characterize changes in muscle recruitment and motor control during rehabilitation (Mosconi et al., 2024). Gait quality is evaluated using kinematic parameters such as stride length and joint mobility, with improvements reflecting enhanced movement efficiency and reduced compensatory strategies (Siviy et al., 2023; Gavrila Laic et al., 2024). Pain severity and analgesic demand are monitored as secondary outcomes, with multimodal analgesia preferred to limit opioid exposure (Berns et al., 2025).

Safety outcomes encompass adverse event surveillance, device-related complications, and treatment adherence to establish the overall risk–benefit profile of exoskeleton-assisted rehabilitation (Morris et al., 2023; Szabo et al., 2023). Telemonitoring platforms further enable remote evaluation of adherence, independence, and quality of life, extending outcome assessment from inpatient care to community settings (Yao et al., 2024; Tsuge et al., 2025).

## 6 Positioning within rehabilitation pathways

### 6.1 Stage-based timing

Exoskeleton integration should align with fracture healing phases, with optimal initiation timing remaining an open question requiring individualized assessment (Hohl et al., 2022). For hip fracture rehabilitation in elderly patients, robotic platforms initiated during early mobilization reduced rehabilitation sessions from 68 to 23 and shortened time to ambulation from 120 to 67 days compared to conventional therapy (Costa et al., 2022). Following proximal humeral fracture surgery, robot-assisted training can commence during the 3-week immobilization period when added to conventional occupational and physical therapy (Nerz et al., 2017). Rehabilitation protocols typically progress through ankle mobility and strengthening exercises, stepping exercises, and weight-bearing balance training, with suggested schedules of two sessions in week one and single sessions in weeks 2–4 (Moseley et al., 2015).

### 6.2 Clinic versus home

Current exoskeleton devices appear better suited for supervised clinical rehabilitation than home use due to device weight, upper extremity support requirements, supervision needs, and limited movement range (Fritz et al., 2019). Although direct orthopedic data for home-based training is lacking, indirect evidence demonstrates that individuals with complete spinal cord injury using home exoskeletons reported satisfaction (D-QUEST 3.7 ± 0.4) but utilized devices primarily for exercise (74%) and social interaction (20%) rather than daily functional activities (van Dijsseldonk et al., 2020). Implementation barriers include calibration time, intensive training requirements, device cost, and setup complexity, raising questions regarding efficacy and cost-effectiveness relative to conventional approaches (Mortenson et al., 2022).

### 6.3 Multidisciplinary coordination

Clinical utilization frameworks emphasize collaborative decision-making regarding device selection, dosage parameters, and progression criteria based on patient deficits and device characteristics (Hohl et al., 2022). Therapists require substantial training to manage the complex human-robot interaction, including patient expectation management and individualized treatment planning (Ehrlich-Jones et al., 2021; Mortenson et al., 2022). Successful implementation demands coordination between orthopedic surgeons determining fixation stability and weight-bearing permissions, physical therapists prescribing exercise progression, and bioengineering support for device optimization (Molteni et al., 2018).

## 7 Key barriers to clinical adoption

### 7.1 System-level limitations

Anthropometric incompatibility represents a fundamental barrier to widespread exoskeleton adoption, with current devices employing a “one-size-fits-all” approach that fails to accommodate diverse body morphologies (Morris et al., 2023). Rigid exoskeletons demonstrate limited adjustability across joint centers and segment lengths, creating misalignments that generate unintended interaction forces and compromise safety (Massardi et al., 2023). Misalignment hazards, particularly rotational deviations around the vertical axis and anteroposterior translations, rank among the most frequently perceived safety concerns in survey-based assessments of exoskeleton users (Massardi et al., 2023). Experimental quantification using instrumented leg simulators reveals that rotational misalignment significantly increases mediolateral knee forces and abduction/adduction torques, while anteroposterior translational misalignment elevates flexion/extension torques during swing phase (Bessler-Etten et al., 2022). These biomechanical perturbations multiply internal joint loads beyond those experienced in well-aligned configurations, warranting careful consideration during device selection and fitting (Bessler-Etten et al., 2022).

Excessive weight constitutes the most frequently cited limitation restricting real-world exoskeleton deployment, with current lower-limb devices ranging from 12 to 27 kg (Fritz et al., 2019; Rodríguez-Fernández et al., 2021). Heavy and bulky designs necessitate upper extremity support through crutches or walkers, increasing energy expenditure and limiting functional independence (Rodríguez-Fernández et al., 2021). Power supply constraints further compound usability challenges, with battery life typically permitting only 2–4 h of continuous operation before requiring recharging (Young and Ferris, 2017). Material selection critically influences overall device mass, with traditional aluminum and steel constructions yielding to carbon fiber composites and advanced polymers to reduce weight while maintaining structural integrity (Hussain et al., 2021). However, these lightweight materials introduce manufacturing complexity and cost escalation that impede clinical accessibility (Hussain et al., 2021).

Biomechanical load transfer errors during exoskeleton-assisted gait pose significant risks for fracture patients with compromised bone integrity (De Carvalho et al., 2025). Computational modeling demonstrates that different approaches to simulating human-robot interactions produce peak compressive forces ranging from 2.98 to 4.66 body weight at the hip, 2.82–5.83 body weight at the knee, and 3.39–3.79 body weight at the ankle, highlighting substantial variability in joint loading patterns (De Carvalho et al., 2025). Adverse events during exoskeleton training predominantly involve skin lesions (pressure ulcers, lacerations) at device-body interface points, with reported rates of 151 events per 1000 h of exposure (McIntosh et al., 2020; Wright et al., 2023). While most adverse events classify as minor or negligible severity, Category II pressure ulcers at contact points occur in approximately 8% of users, requiring protocol modifications or temporary training cessation (Bach Baunsgaard et al., 2018; Wright et al., 2023). Long-term safety data remain limited, with 97% of existing studies measuring only short-term effects and lacking evidence regarding cumulative musculoskeletal adaptations or degenerative changes from prolonged use (Kranenborg et al., 2023).

### 7.2 Human and clinical adoption barriers

#### 7.2.1 User adherence and comfort

Device weight, setup complexity, and physical discomfort represent primary barriers to patient adherence with exoskeleton rehabilitation (Morris et al., 2023). Systematic reviews identify six critical user-centered limitations: safety concerns, one-size-fits-all design approaches, ease of device use, weight and placement, cost, and device appearance (Morris et al., 2023). Satisfaction analyses reveal that while users rate exoskeletons favorably for safety, efficacy, and comfort (mean satisfaction scores 31.3 ± 5.70 out of 40 for patients), the worst-rated aspects requiring optimization include ease of adjustment, size and weight, and ease of use (Cumplido-Trasmonte et al., 2023). Implementation studies document high prevalence of adverse events including skin issues and falls, alongside frequent device malfunctions, as recurrent barriers to sustained clinical use (Charette et al., 2023).

Patient expectations frequently exceed actual therapeutic benefits, creating a critical gap between perceived and evidence-based outcomes that clinicians must actively manage (Louie et al., 2022). Qualitative analyses derived from indirect stroke rehabilitation evidence demonstrate that stroke patients express more optimism toward exoskeleton benefits than therapists, attributing greater opportunity and benefit to device use during rehabilitation (Louie et al., 2022). However, therapists identify the need to balance actual versus perceived benefits and manage patient expectations as essential implementation considerations (Mortenson et al., 2022). Perceived usefulness in terms of time and effort savings represents the primary factor influencing therapist willingness to use exoskeletons, with acceptance increasing among those with prior technology experience while distrust decreases (Luciani et al., 2023).

Device appearance concerns patients specifically, though this factor does not affect therapist adoption decisions (Morris et al., 2023).

Robotic assistance can paradoxically disrupt normal motor patterns and increase gait variability in ways that may impede motor learning (Berger et al., 2019; Marco et al., 2023). Healthy volunteers demonstrate increased gait variability during robot-assisted walking accompanied by increased sensorimotor brain activity, suggesting that robotic guidance may disrupt the cortical network of automated walking (Berger et al., 2019). Single-session exoskeleton training modulates complexity in movement patterns, increasing regularity of body oscillations while causing loss of alternating muscle activation during the gait cycle (Marco et al., 2023). Muscle synergy analyses reveal that while some stroke survivors adapt to exoskeletal assistance by altering synergy patterns toward normal, others experience undesired synergy changes from adapting to the device’s mechanical properties (Rinaldi et al., 2020). The risk exists that complete robotic assistance may induce passive motor system engagement, though controlled studies demonstrate that exoskeleton training preserves muscle coordination complexity without significantly altering neuromuscular patterns when properly implemented (Zhu et al., 2021).

### 7.3 Economic and regulatory constraints

#### 7.3.1 Cost-effectiveness

Robotic exoskeleton rehabilitation shows variable cost-effectiveness depending on clinical utilization and organizational models (Pinto et al., 2020; Fernandez-Garcia et al., 2021; Shankar et al., 2025). Drawing on indirect economic models from Singaporean stroke rehabilitation, exoskeleton therapy achieved an incremental cost-effectiveness ratio of US$28,260 per QALY for severely impaired patients, indicating potential cost-effectiveness (Shankar et al., 2025). In contrast, the RATULS trial in the UK reported £1,601 higher costs without QALY gains, with only a 19% probability of cost-effectiveness at conventional thresholds (Fernandez-Garcia et al., 2021). Similarly, indirect evidence from budget analyses for spinal cord injury suggest partial session use can reduce annual training costs, while staffing models—such as one therapist supervising multiple patients—can yield substantial savings, whereas 1:1 ratios substantially increase costs (Pinto et al., 2020). Mixed robotic and conventional protocols can further reduce expenses compared with fully conventional therapy (Gower et al., 2024).

#### 7.3.2 Reimbursement uncertainty

Uncertain reimbursement pathways remain a key barrier to adoption, as device availability, pricing, and approval for home use vary regionally, and intensive training and supervision requirements raise questions about relative efficacy (Mortenson et al., 2022). FDA approval for medical exoskeletons typically follows the 510(k) pathway for moderate-risk Class II devices, demonstrating equivalence rather than full clinical efficacy, while the European Medical Device Regulation mandates stricter equivalence evidence and post-market data collection (Darrow et al., 2021; Hohl et al., 2022). These divergent regulatory frameworks complicate international market access and contribute to implementation delays.

#### 7.3.3 Motor adaptation and dependency risks

Excessive robotic assistance may impede motor learning by reducing active patient engagement and limiting neuroplastic adaptations (Rodgers et al., 2019; Hasson et al., 2023). The RATULS trial showed improvements in impairment measures without corresponding functional gains, highlighting that repetitive automated movements may not generalize to daily activities. Optimal human-robot interaction remains unclear, with therapist-in-the-loop approaches potentially enhancing motor adaptation more effectively than full automation (Hasson et al., 2023). Properly implemented exoskeleton training can increase cortical excitability and frontoparietal connectivity, but meaningful functional outcomes require careful patient selection, task-specific practice, and integration with conventional therapy to avoid dependency on robotic support (Calabrò et al., 2018; Fan et al., 2025).

### 7.4 Evidence-based strategies for clinical adoption

Bridging the gap between prototype development and real-world clinical adoption requires actionable, evidence-based strategies to overcome barriers in cost, regulation, and workflow. Economically, transitioning from traditional capital purchasing to ‘Device-as-a-Service’ (DaaS) or leasing models can significantly lower the initial acquisition threshold for healthcare facilities. Regulatory pathways must be streamlined by establishing standardized biomechanical safety endpoints—such as quantifiable peak interfragmentary strain limits—to facilitate smoother FDA and CE clearances for autonomous systems. Operationally, the seamless integration of exoskeleton telemetric data (e.g., gait metrics, loading cycles) directly into Electronic Health Records (EHR) is critical. Furthermore, within high-volume orthopedic departments, establishing dedicated ‘rehabilitation engineer’ roles or specialized technician workflows can ensure that complex device setup and parameter tuning do not overburden existing clinical staff, thereby making daily clinical adoption highly sustainable.

## 8 Future directions

The evolution of smart exoskeletons for post-fracture recovery is moving toward systems that are more responsive to both the mechanical needs of the healing bone and the biological state of the patient. Future research and development should focus on three primary pillars to bridge the gap between laboratory innovation and routine clinical use.

### 8.1 Toward lightweight, adaptive, assist-as-needed systems

Future designs must prioritize the transition from rigid, heavy structures to lightweight, compliant systems to improve user adherence and functional independence.

#### 8.1.1 Material Innovation

Further integration of carbon fiber composites and advanced polymers is essential to reduce device mass from the current 12–27 kg range without compromising structural integrity.

#### 8.1.2 Soft Actuation

The adoption of flexible or pneumatic actuators can better accommodate nonlinear joint mechanics and improve comfort.

#### 8.1.3 Intelligent Assistance

Control strategies will increasingly utilize “assist-as-needed” (AAN) algorithms. These systems use real-time muscle activation (sEMG) and force sensors to quantify residual function, providing only the minimum torque necessary to complete a movement. This approach prevents passive engagement and encourages the voluntary effort required for neuromuscular recovery.

### 8.2 Data-driven personalization and digital twins

**FIGURE 5:**
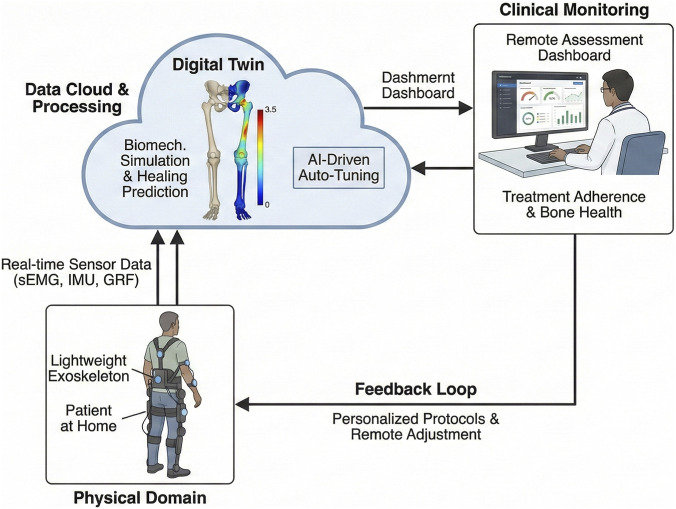
Digital Twin-Driven Tele-Rehabilitation Ecosystem. This future-oriented framework integrates physical rehabilitation with virtual musculoskeletal modeling. Real-time sensor data (sEMG, IMU, GRF) from the patient’s home are transmitted to a cloud-based Digital Twin for biomechanical simulation and predictive healing analysis. AI algorithms perform autonomous parameter tuning, while clinicians monitor bone health and treatment adherence remotely. This ecosystem transitions fracture recovery from clinical sessions to personalized, data-driven tele-rehabilitation in community settings.

The next-generation of fracture rehabilitation will likely leverage Digital Twin (DT) technology to create virtual representations of a patient’s musculoskeletal system and healing fracture (Figure 5).

#### 8.2.1 Biomechanical simulation

By integrating real-time sensor data (IMU, GRF, and EMG) into computational models, clinicians can predict interfragmentary strain and peak compressive forces at the fracture site.

#### 8.2.2 Predictive healing models

Digital twins can simulate various loading protocols—such as the 2%–10% beneficial interfragmentary strain range—to determine the optimal timing for progressive weight-bearing without risking fixation failure (Yao et al., 2024; Li et al., 2026a).

#### 8.2.3 AI-driven tuning

Machine learning algorithms will enable autonomous, patient-specific parameter tuning, reducing the current burden of manual calibration and allowing the device to adapt to subtle changes in gait or fatigue in real time.

### 8.3 Multimodal rehabilitation integration

To maximize therapeutic outcomes, exoskeletons should not operate in isolation but as part of a multimodal, integrated care pathway.

#### 8.3.1 Telemonitoring and remote care

Integration with cloud-based platforms will allow for continuous remote assessment of bone health, treatment adherence, and adverse events (such as skin lesions) in community settings.

#### 8.3.2 Hybrid therapy

Combining robotic assistance with conventional physical therapy and other technologies—such as functional electrical stimulation (FES)—may enhance cortical excitability and frontoparietal connectivity more effectively than either method alone.

#### 8.3.3 Standardized clinical frameworks

Future efforts must establish multidisciplinary protocols that coordinate surgeon-defined fixation stability with therapist-led exercise progression, aiming to align technology with the biological requirements of bone regeneration.

## 9 Conclusion

### 9.1 Synthesis of current technological advances and clinical evidence

Sensor-rich, powered exoskeletons represent a shift in orthopedic rehabilitation, enabling adaptive, assist-as-needed support. While generally safe and capable of improving mobility and hospital stay in select contexts, clinical evidence remains limited and heterogeneous, and consistent benefits over conventional therapy for complex fractures are not yet established.

### 9.2 Practical recommendations for clinicians and developers

Clinicians should assess fixation stability, manage patient expectations, and monitor for device-related adverse events. Developers should prioritize lightweight, modular designs, reduce setup complexity, and implement standardized safety protocols to facilitate adoption.

### 9.3 Priority research questions and roadmap toward fully personalized, data-driven fracture rehabilitation with smart exoskeletons

Future studies should define optimal mechanobiological loading, evaluate long-term efficacy and cost-effectiveness, and advance home-based, user-friendly systems to extend rehabilitation beyond the clinic.
